# Two Basal Cell Carcinomas of the Axillae: A Metastasis or an Independent Development?

**DOI:** 10.1155/2015/637292

**Published:** 2015-03-15

**Authors:** Yuichiro Endo, Yoshiki Miyachi

**Affiliations:** Department of Dermatology, Graduate School of Medicine, Kyoto University, Kyoto 606-8507, Japan

## Abstract

Cutaneous basal cell carcinoma (BCC) is a common malignancy amongst the elderly. BCC rarely metastasises, and fewer than 300 cases of metastatic BCC have been reported in the literature. Here, we report a case of sequentially developed BCCs located adjacent to each other. We discuss that these BCCs were considered to have developed independently rather than due to metastasis, after referencing previous studies on metastatic BCC.

## 1. Case Report

**Figure 1:**
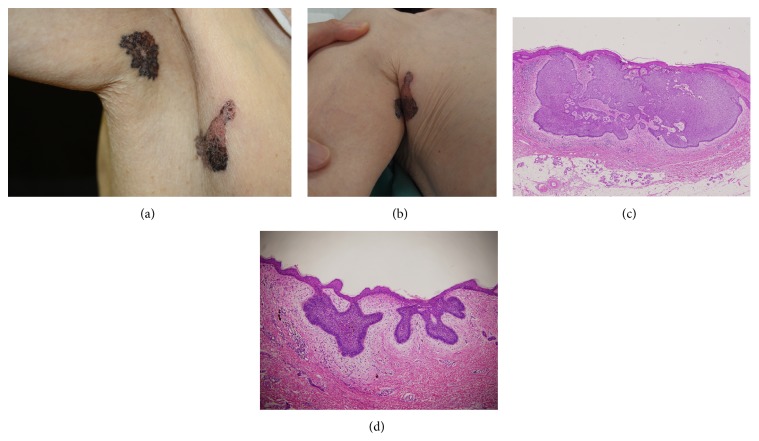
(a) Clinical appearance of basal cell carcinomas in the right axillae. (b) Both tumours contacted each other when the right shoulder joint was adducted. (c) Histological findings of the tumour that appeared first and was located on the axillae side. Atypical basaloid cells small forming isolated islands of tumour attached to the basement of the epidermis (Haematoxylin and Eosin stain, original magnification ×200). (d) The histopathological findings of the second one were the same as the first one. Tumour thickness was 0.55 mm (Haematoxylin and Eosin stain, original magnification ×100).

An 80-year-old woman presented at our department with two tumours in the right axillary area. A medical interview revealed that the tumour located on the axillae appeared first. The patient reported that the tumour was sometimes accompanied by excoriation and serous discharge. Four years after the appearance of the first tumour, the second one developed on the right side of the chest and contacted the first tumour when the right shoulder joint was adducted (Figures 1(a) and 1(b)). Physical examinations showed no lymph node metastasis. Surgical excisions with 5 mm margins were conducted. The patient was not exposed to radioactive materials or radiation therapy. Histopathological examinations revealed that both BCCs were superficial and had multifocal tumour lesions (Figures 1(c) and 1(d)). Surgical margins were negative and not continuum. Orthopantomography and physical examinations showed no evidence of Gorlin syndrome.

## 2. Discussion

BCC is the most prevalent form of skin cancer which develops in the basal parts of the epidermis and rarely metastasizes [1–3]. Pathological findings of BCC are characterised by its local invasiveness and downgrowth of basaloid cells with peripheral palisading and cleft formation [3]. BCC rarely metastasizes, with an estimated incidence of 0.0028–0.55% [2].

The routes of metastasis of skin cancers are classified into the following four categories: lymphatic, haematogenous, disseminative, and direct invasion [3]. Direct invasion is rare and occurs when a tumour has contact with a possible metastatic site, for example, carcinoma “en cuirasse” [4]. However, cutaneous squamous cell carcinomas on the upper and lower lips, which were formally considered contact metastases, are currently not regarded as an instance of the phenomenon. This is because the upper and lower lips merely share the same risk factors for cancer generation, such as sunlight exposure or smoking, and the concurrence is not a result of metastasis but simultaneous development.

The diagnostic criteria of metastatic BCC were advocated by Lattes and Kessler [5]. According to their descriptions [5], the following three conditions need to be met for diagnosis of metastatic BCC: (1) the primary tumour is cutaneous and does not originate from mucosal or glandular tissue; (2) the primary tumour and metastatic lesion have identical histopathological features; and (3) metastases are clearly distant from the primary tumour and do not result from direct invasion. In our case, the BCCs were pathologically classified as superficial. Although these BCCs could be regarded as metastases in light of the criteria, metastatic BCC is very rare. The reason for this is that the posterior BCC showed multifocal growths, which pathologically support independent occurrence of the tumours. Second, knowledge of carcinogenesis supports concurrence. BCC usually metastasizes to the lymph nodes and less frequently to the skin and other organs. Metastases from BCC are rare because BCC depends on the surrounding stroma, as shown by the inability to transplant BCC cells to other humans without associated stroma [6].

Then, how do we interpret this phenomenon? In a survey of 732 patients with BCC in Japan, 52 (7.1%) showed multiple lesions and >50% of these lesions were one-sided and located close to each other [7]. One possible explanation is postzygotic somatic mutation [7]. Namely, gene mutations that occur in cells after fertilisation (postzygotic) can result in mosaicism, which is defined as the presence of a mutation, deletion, or chromosomal abnormality in some cell groups. Areas with abnormal cells are prone to develop multiple tumours of the same kind. Although no easy method to detect such mutations is currently available, such a modality is needed to clarify the pathogenesis of multiple BCCs.
